# A Microsatellite Guided Insight into the Genetic Status of Adi, an Isolated Hunting-Gathering Tribe of Northeast India

**DOI:** 10.1371/journal.pone.0002549

**Published:** 2008-07-02

**Authors:** S. Krithika, Suvendu Maji, T. S. Vasulu

**Affiliations:** Biological Anthropology Unit, Indian Statistical Institute, Kolkata, India; Centre for DNA Fingerprinting and Diagnostics, India

## Abstract

Tibeto-Burman populations of India provide an insight into the peopling of India and aid in understanding their genetic relationship with populations of East, South and Southeast Asia. The study investigates the genetic status of one such Tibeto-Burman group, Adi of Arunachal Pradesh based on 15 autosomal microsatellite markers. Further the study examines, based on 9 common microsatellite loci, the genetic relationship of Adi with 16 other Tibeto-Burman speakers of India and 28 neighboring populations of East and Southeast Asia. Overall, the results support the recent formation of the Adi sub-tribes from a putative ancestral group and reveal that geographic contiguity is a major influencing factor of the genetic affinity among the Tibeto-Burman populations of India.

## Introduction

Northeast India has always been a hotspot for population geneticists due to its unique and strategic geographic location and the presence of linguistically, culturally and demographically diverse populations practicing varied occupations (from hunting-gathering to settled agriculture) [1]–[3]. Due to their relative geophysical isolation (flanked by the Eastern Himalayas in the northern and the Bay of Bengal in the southern region), leading to limited external gene flow, these diverse populations retain a unique population structure which in turn is expected to reflect in their gene pools.

This region exhibits linguistic diversity (represented by Tibeto-Burman, Austro-Asiatic and Indo-European language families) which can be attributed to diverse socio-cultural influences, extensive population interactions and putative long history of migrations experienced by the region in the past [4]–[5]. The Tibeto-Burman speaking populations predominate the region, about 2% of the total Indian population [6], representing a significant component of the biological diversity of the peopling of India. They exhibit vast diversity with respect to culture, language, subsistence economy and population structure variables like size, growth, distribution, marriage patterns and degree of endogamy [2], [7]–[8]. These populations are of significance in understanding the peopling of India and in comprehending the relationship prevailing among the regional populations, as well as the relationship of these populations with the neighboring East/Southeast Asian groups to whom they are morphologically, ethno-historically and linguistically affiliated [2], [4]–[5], [7]–[8]. In view of their importance, many researchers have earlier attempted, using classical and molecular genetic markers, to address various population genetic issues pertaining to these regional groups. The studies were however sporadic and restricted to only few regional populations [9]–[20]. In this regard, the Tibeto-Burman speaking populations inhabiting the easternmost tip of northeast India, Arunachal Pradesh, (sharing the international border between India and Bhutan, Tibet, Myanmar) were hardly dealt with and hence there exist a dearth of population genetic studies in this region [21]–[24]. However, Arunachal Pradesh is of importance from a population genetic perspective, as this region has experienced cultural contacts and population interactions due to multitude waves of migration, during different periods, from the adjoining regions [25].

Arunachal Pradesh (situated between latitude 26°30′N and 29°30′N and longitude 91°30′E and 97°30′E) is the abode of 26 major Tibeto-Burman speaking tribes and 110 sub-tribes and minor tribes [25], majority claiming their descent from the Tibetan region during different time periods (evident from the available ethno-historical accounts and folklore tradition). One of the largest tribe of the region is Adi, a collective tribe distributed in the temperate and sub-tropical regions within the districts of West Siang, East Siang, Upper Siang, Upper Subansiri and Dibang Valley in central Arunachal Pradesh [25]–[26]. They share similar physical features of that of East Asian populations and speak Adi dialects which belong to North-Assam branch of Tibeto-Burman sub-linguistic family [27]. The ethno-history suggests their origin from southern regions of Tibet (China) and traces their migration and settlement history of their ancestors (the ‘Tani’ group) at different time periods during about 5^th^–7^th^ century AD [26], [28]–[30]. There are about 12 sub-tribes of Adi, categorized under two major clusters based on their dialect, culture, ethno-historical migration and distribution along the Siang river valley. While one cluster consists of the Minyong, Padam, Shimong, Milan, Pasi, Panggi and Komkar sub-tribes, the other includes Gallong, Ramo, Bokar, Pailobo and Bori sub-tribes. Among these 12 sub-tribes, Minyong and Padam are numerically large (about several thousands), the rest being small (in hundreds) [29]–[30] and live in isolation in upper mountain regions. Their ethno-historical records suggest that these sub-tribes are as a resultant of fission-fusion processes due to inter tribal war fares [26], [28]. Some of the remotely located (in mountainous terrains) Adi sub-populations practice hunting-gathering, while some others located over plain lands, in close proximity to urban area, practice settled agriculture. These wide variations in population structure, coupled with the vast diversity in their culture including religious beliefs, customs, dialect, house types, food habits, dress and ornaments' pattern and demography, render importance to these groups from a population genetic perspective [29]–[31].

**Figure 1:**
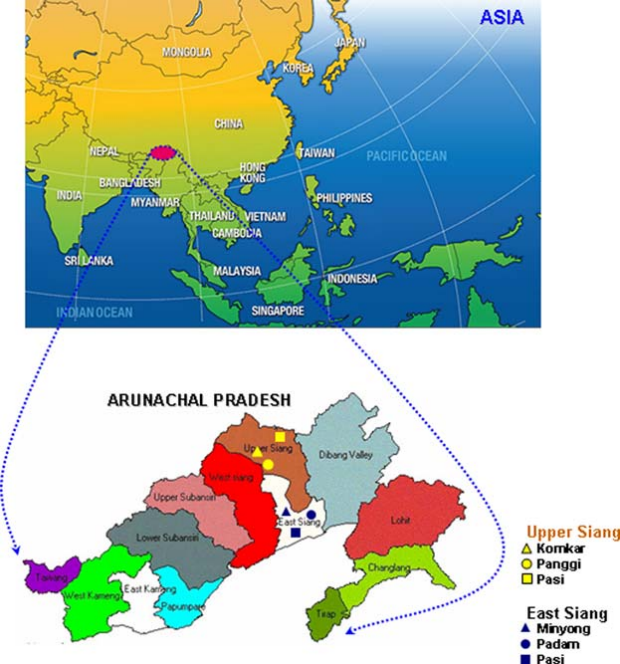
Map of Arunachal Pradesh showing the geographical distribution of the studied Adi sub-tribes.

The present study, perhaps for the first time, investigates the genetic status of six sub-groups of Adi tribe (Figure 1) based on a set of 15 autosomal microsatellite (STR) markers. The study explores the genetic status of the Adi sub-tribes and examines their genetic relationship with the neighboring populations of India and East/Southeast Asia to whom they are affiliated [17].

## Results

Adi tribe, a Tibeto-Burman speaking population of northeast India, is of importance in understanding the genetic affinity among the Tibeto-Burman speaking tribes of India and neighboring populations of East/Southeast Asia. The results of the genetic affinity and diversity among Adi sub-groups, their differentiation as well as sub structuring have been presented in this study. Also, the genetic relationship of Adi with neighboring Tibeto-Burman populations of north and northeast India as well as with the linguistically divergent populations of East/Southeast Asia have been discussed.

### Extent of microsatellite diversity

The allele frequency distribution of the 15 STR loci among the studied six Adi sub-groups has been previously reported [32]–[34]. A summary of the descriptive statistics including the locus-wise heterozygosity (*h*), probability of homozygosity (*P*), Exact test (ET) and Likelihood ratio test (LR) (performed to check for departure from Hardy-Weinberg equilibrium) values are shown in Table 1. Adi Pasi-Upper exhibit departure from Hardy-Weinberg Equilibrium (HWE) at six loci (D8S1179, CSF1P0, D3S1358, THO1, vWA and TPOX) while Adi Panggi show departure at four loci (TPOX, D19S433, FGA, D8S1179). Padam is the only population where all loci were found to be in equilibrium. The average heterozygosity values that reflects the extent of genetic diversity (within-population heterogeneity) exhibit a narrow range between 0.7404 (Adi Panggi) and 0.7810 (Adi Pasi-Upper). The locus-wise G_ST_ values show least differentiation for the vWA locus (0.9398%) and a high degree of differentiation in case of D3S1358 locus (3.8685%). The average G_ST_ value is observed to be 2.34% indicative of the lower degree of genetic differentiation among Adi populations.

**Table 1:** Locus-wise and population-wise descriptive statistics among the studied populations, based on 15 STR loci

Locus	Populations	Statistical Parameters			
		*ET*	*LR*	*h*	*p*
**D5S818**	***Lower Pasi***	0.4850	0.5300	0.7650	0.1560
	***Upper Pasi***	0.2335	0.0010	0.7910	0.2882
	***Minyong***	0.4480	0.4265	0.5200	0.0012
	***Panggi***	0.2655	0.1405	0.8595	0.0242
	***Komkar***	0.2935	0.1595	0.8226	0.2424
	***Padam***	0.8625	0.9055	0.8043	0.8955
**FGA**	***Lower Pasi***	0.4330	0.7170	0.7950	0.0001
	***Upper Pasi***	0.1640	0.0740	0.8060	0.0142
	***Minyong***	0.0185	0.0130	0.8125	0.4497
	***Panggi***	0.0065	0.0045	0.8451	0.9704
	***Komkar***	0.6670	0.8715	0.7674	0.0864
	***Padam***	0.2540	0.3510	0.6591	0.0000
**D8S1179**	***Lower Pasi***	0.0020	0.0000	0.8270	0.4880
	***Upper Pasi***	0.0000	0.0000	0.9170	0.0022
	***Minyong***	0.0225	0.0190	0.8667	0.2039
	***Panggi***	0.0000	0.0000	0.8636	0.0545
	***Komkar***	0.0090	0.0075	0.8730	0.2908
	***Padam***	0.0345	0.0225	0.9200	0.0108
**D21S11**	***Lower Pasi***	0.6430	0.8550	0.8030	0.3550
	***Upper Pasi***	0.2710	0.0390	0.6670	0.0016
	***Minyong***	0.6005	0.5150	0.7083	0.6842
	***Panggi***	0.0000	0.0000	0.8818	0.0024
	***Komkar***	0.3425	0.2000	0.9032	0.1106
	***Padam***	0.3420	0.5205	0.8200	0.8597
**D7S820**	***Lower Pasi***	0.5300	0.7540	0.6200	0.0002
	***Upper Pasi***	0.2530	0.2660	0.7500	0.4362
	***Minyong***	0.0825	0.1590	0.6364	0.1424
	***Panggi***	0.1490	0.3175	0.6628	0.1409
	***Komkar***	0.9930	0.6981	0.6981	0.5583
	***Padam***	0.1225	0.0955	0.7727	0.7349

Locus	Populations	Statistical Parameters			
		*ET*	*LR*	*h*	*p*
**CSF1PO**	***Lower Pasi***	0.1970	0.1800	0.6700	0.3820
	***Upper Pasi***	0.0010	0.0020	0.5320	0.0054
	***Minyong***	0.0715	0.2275	0.4545	0.1477
	***Panggi***	0.0795	0.0235	0.6667	0.7589
	***Komkar***	0.9115	0.8145	0.7143	0.4788
	***Padam***	0.0640	0.2165	0.5217	0.2348
**D3S1358**	***Lower Pasi***	0.2550	0.4090	0.6960	0.6980
	***Upper Pasi***	0.0020	0.0020	0.8160	0.0250
	***Minyong***	0.7245	0.5775	0.6207	0.1423
	***Panggi***	0.0445	0.0250	0.8073	0.0130
	***Komkar***	0.9950	0.9775	0.7619	0.7348
	***Padam***	0.7700	0.7805	0.6800	0.7600
**THO1**	***Lower Pasi***	0.4280	0.0050	0.6000	0.6970
	***Upper Pasi***	0.0005	0.0000	0.7520	0.0022
	***Minyong***	0.0030	0.0025	0.7241	0.2641
	***Panggi***	0.4535	0.3315	0.6273	0.0294
	***Komkar***	0.0540	0.0280	0.6190	0.4228
	***Padam***	0.6985	0.4965	0.7000	0.0972
**D13S317**	***Lower Pasi***	0.7430	0.8390	0.7990	0.7540
	***Upper Pasi***	0.4810	0.0800	0.6880	0.0018
	***Minyong***	0.8650	0.9365	0.6842	0.4553
	***Panggi***	0.1195	0.0760	0.7624	0.3300
	***Komkar***	0.4430	0.4165	0.8131	0.5465
	***Padam***	0.9860	0.9685	0.7708	0.8012
**D16S5139**	***Lower Pasi***	0.0320	0.0710	0.7270	0.3900
	***Upper Pasi***	0.2910	0.0670	0.7600	0.2645
	***Minyong***	0.3145	0.2775	0.7778	0.8607
	***Panggi***	0.0190	0.0325	0.6875	0.6560
	***Komkar***	0.0515	0.0945	0.6393	0.4217
	***Padam***	0.6600	0.6690	0.7500	0.6301

Locus	Population	Statistical Parameters			
		*ET*	*LR*	*h*	*p*
**D2S1338**	***Lower Pasi***	0.1770	0.3100	0.7550	0.0002
	***Upper Pasi***	0.2630	0.2040	0.7050	0.0000
	***Minyong***	0.4355	0.2925	0.9545	0.2730
	***Panggi***	0.1935	0.2295	0.7561	0.0608
	***Komkar***	0.0925	0.0830	0.8750	0.3816
	***Padam***	0.7350	0.8775	0.7872	0.2614
**D19S433**	***Lower Pasi***	0.0010	0.3060	0.7950	0.0187
	***Upper Pasi***	0.1220	0.0040	0.8950	0.0067
	***Minyong***	0.0160	0.0145	0.7037	0.3349
	***Panggi***	0.0000	0.0000	0.9450	0.0329
	***Komkar***	0.0000	0.0000	0.8730	0.2753
	***Padam***	0.7165	0.6530	0.8600	0.7637
**Vwa**	***Lower Pasi***	0.2820	0.0660	0.8150	0.9100
	***Upper Pasi***	0.0000	0.0000	0.8450	0.0265
	***Minyong***	0.0150	0.0225	0.6154	0.3347
	***Panggi***	0.0035	0.0005	0.8455	0.1544
	***Komkar***	0.0000	0.0000	0.8730	0.0388
	***Padam***	0.1595	0.0485	0.8600	0.1999
**TPOX**	***Lower Pasi***	0.2830	0.0650	0.5550	0.0014
	***Upper Pasi***	0.0000	0.0000	0.8120	0.0105
	***Minyong***	0.0535	0.0320	0.6667	0.7009
	***Panggi***	0.0000	0.0000	0.8073	0.0233
	***Komkar***	0.7945	0.3990	0.6667	0.4691
	***Padam***	0.1035	0.4035	0.7941	0.9360
**D18S51**	***Lower Pasi***	0.0010	0.0010	0.6800	0.0015
	***Upper Pasi***	0.0980	0.2080	0.6810	0.0391
	***Minyong***	0.1215	0.0575	1.0000	0.1945
	***Panggi***	0.2400	0.3650	0.6029	0.7448
	***Komkar***	0.6645	0.6210	0.7143	0.6395
	***Padam***	0.4120	0.4035	0.7941	0.9360

ET: Exact Test; LR: Likelihood Ratio Test (ET and LR tests were done to test for departure from Hardy-Weinberg equilibrium); h: average heterozygosity; p: Probability of homozygosity

### Genetic differentiation and sub-structuring among Adi tribe

The locus-wise results of the exact test of population differentiation are shown in Table 2. Pair wise comparison of the sub-tribes of Adi reveal most significant difference (at 12 loci: D5S818, FGA, D21S11, D7S820, D3S1358, THO1, D13S317, D16S539, vWA, D18S818, D2S1338, D19S433) between Adi Pasi-Upper and Adi Panggi. Adi Pasi-Upper also shows significant differences at 9 loci with Adi Pasi-Lower (FGA, D7S820, D3S1358, THO1, D13S317, vWA, TPOX, D2S1338, D19S433) and at 8 loci with Adi Komkar (D21S11, D7S820, D3S1358, D13S317, D16S539, TPOX, D18S818, D19S433). Least significant difference is shown by Adi Padam with Adi Pasi-Lower (at locus D2S1338) and Adi Komkar (at locus D7S820). Overall, Adi Pasi-Upper shows significant difference, at loci D7S820 and D13S317, with all the other study populations. Among the analyzed 15 STR loci, D8S1179 shows no significant difference among the Adi sub-tribes and also CSF1PO and D5S818 show significant difference only in one pair of populations (between Adi Pasi-Lower–Adi Panggi and Adi Pasi-Upper –Adi Panggi respectively).

**Table 2:** Pair-wise comparison of studied populations, at the analyzed loci, to investigate the extent of population differentiation. *Significant values are in bold*

	D5S818	FGA	D8S1179	D21S11	D7S820	CSF1PO	D3S1358	THO1	D13S317	D16S539	vWA	TPOX	D18S818	DD2S1338	D19S433
**Adi Pasi-Upper vs.**															
Adi Pasi-Lower	0.06465	**0.01800**	0.49215	0.22920	**0.00185**	0.13430	**0.03195**	**0.00010**	**0.00000**	0.33640	**0.00000**	**0.01660**	0.80665	**0.00000**	**0.00035**
Adi Minyong	0.34695	0.39540	0.67800	0.18815	**0.01100**	0.20185	0.41870	0.13835	**0.00000**	0.79705	0.06930	0.18015	0.06785	0.81240	0.08020
Adi Panggi	**0.03785**	**0.03935**	0.85865	**0.01665**	**0.00000**	0.05485	**0.00000**	**0.00775**	**0.00085**	**0.04885**	**0.03160**	0.06360	**0.00010**	**0.00650**	**0.00000**
Adi Komkar	0.10615	0.20650	0.68935	**0.01295**	**0.00005**	0.59755	**0.03145**	0.38530	**0.00070**	**0.00090**	0.12155	**0.01655**	**0.00830**	0.07495	**0.00000**
Adi Padam	0.27425	0.75485	0.55545	0.12455	**0.03400**	0.21260	0.66870	0.14005	**0.00530**	0.66445	**0.00295**	0.08100	0.14690	0.65955	**0.00035**
**Adi Pasi-Lower vs.**															
Adi Pasi-Upper	0.06465	**0.01800**	0.49215	0.22920	**0.00185**	0.13430	**0.03195**	**0.00010**	**0.00000**	0.33640	**0.00000**	**0.01660**	0.80665	**0.00000**	**0.00035**
Adi Minyong	0.09725	**0.00350**	0.52945	0.06310	**0.02805**	0.18285	0.46815	**0.00015**	**0.00000**	0.39905	**0.00025**	0.38315	0.09075	**0.00000**	0.07220
Adi Panggi	0.30200	0.07095	0.10510	0.12200	0.90380	**0.02310**	**0.00000**	0.10955	0.11970	0.51925	**0.02920**	**0.03105**	**0.02480**	**0.00000**	**0.00035**
Adi Komkar	0.14050	0.07890	0.36885	**0.01195**	0.51530	0.89125	**0.00045**	0.22085	0.69185	**0.01010**	0.65870	0.61110	0.16790	**0.00000**	**0.00000**
Adi Padam	0.52230	0.09780	0.42775	0.05375	0.09885	0.40990	0.84910	0.37805	0.53015	0.90070	0.24215	0.96020	0.27535	**0.00000**	0.85170
**Adi Minyong vs.**															
Adi Pasi-Upper	0.34695	0.39540	0.67800	0.18815	**0.01100**	0.20185	0.41870	0.13835	**0.00000**	0.79705	0.06930	0.18015	0.06785	0.81240	0.08020
Adi Pasi-Lower	0.09725	**0.00350**	0.52945	0.06310	**0.02805**	0.18285	0.46815	**0.00015**	**0.00000**	0.39905	**0.00025**	0.38315	0.09075	**0.00000**	0.07220
Adi Panggi	0.23235	**0.00020**	0.70550	**0.00005**	0.16625	0.07645	**0.00110**	**0.00145**	**0.00000**	0.35350	0.47035	0.05580	**0.00040**	**0.02730**	0.05430
Adi Komkar	0.27105	0.05525	0.57185	**0.00745**	0.06130	0.22985	0.07720	0.05715	**0.00045**	**0.03595**	0.05295	0.06420	**0.03215**	0.05015	0.08445
Adi Padam	0.21875	0.36595	0.74540	**0.01520**	0.28305	0.29460	0.73770	0.05390	**0.01475**	0.77980	**0.00110**	0.35780	**0.01830**	0.80295	0.27240
**Adi Panggi** **vs.**															
Adi Pasi-Upper	**0.03785**	**0.03935**	0.85865	**0.01665**	**0.00000**	0.05485	**0.00000**	**0.00775**	**0.00085**	**0.04885**	**0.03160**	0.06360	**0.00010**	**0.00650**	**0.00000**
Adi Pasi-Lower	0.30200	0.07095	0.10510	0.12200	0.90380	**0.02310**	**0.00000**	0.10955	0.11970	0.51925	**0.02920**	**0.03105**	**0.02480**	**0.00000**	**0.00035**
Adi Minyong	0.23235	**0.00020**	0.70550	**0.00005**	0.16625	0.07645	**0.00110**	**0.00145**	**0.00000**	0.35350	0.47035	0.05580	**0.00040**	**0.02730**	0.05430
Adi Komkar	0.32770	**0.02875**	0.34845	**0.00000**	0.62605	0.79735	0.09740	0.84680	0.16955	**0.02050**	0.28225	0.28285	**0.01225**	0.42580	**0.03550**
Adi Padam	0.60480	0.22565	0.64190	**0.03165**	0.23055	0.94185	**0.04030**	0.42670	0.40295	0.88225	**0.03120**	0.41840	**0.01715**	0.29115	0.27540
**Adi Komkar vs.**															
Adi Pasi-Upper	0.10615	0.20650	0.68935	**0.01295**	**0.00005**	0.59755	**0.03145**	0.38530	**0.00070**	**0.00090**	0.12155	**0.01655**	**0.00830**	0.07495	**0.00000**
Adi Pasi-Lower	0.14050	0.07890	0.36885	**0.01195**	0.51530	0.89125	**0.00045**	0.22085	0.69185	**0.01010**	0.65870	0.61110	0.16790	**0.00000**	**0.00000**
Adi Minyong	0.27105	0.05525	0.57185	**0.00745**	0.06130	0.22985	0.07720	0.05715	**0.00045**	**0.03595**	0.05295	0.06420	**0.03215**	0.05015	0.08445
Adi Panggi	0.32770	**0.02875**	0.34845	**0.00000**	0.62605	0.79735	0.09740	0.84680	0.16955	**0.02050**	0.28225	0.28285	**0.01225**	0.42580	0.03550
Adi Padam	0.50350	0.19375	0.33005	0.78170	**0.00520**	0.70790	0.24580	0.75995	0.96405	0.25815	0.08190	0.70330	0.16685	0.17405	0.18010
**Adi Padam vs.**															
Adi Pasi-Upper	0.27425	0.75485	0.55545	0.12455	**0.03400**	0.21260	0.66870	0.14005	**0.00530**	0.66445	**0.00295**	0.08100	0.14690	0.65955	**0.00035**
Adi Pasi-Lower	0.52230	0.09780	0.42775	0.05375	0.09885	0.40990	0.84910	0.37805	0.53015	0.90070	0.24215	0.96020	0.27535	**0.00000**	0.85170
Adi Minyong	0.21875	0.36595	0.74540	**0.01520**	0.28305	0.29460	0.73770	0.05390	**0.01475**	0.77980	**0.00110**	0.35780	**0.01830**	0.80295	0.27240
Adi Panggi	0.60480	0.22565	0.64190	**0.03165**	0.23055	0.94185	**0.04030**	0.42670	0.40295	0.88225	**0.03120**	0.41840	**0.01715**	0.29115	0.27540
Adi Komkar	0.50350	0.19375	0.33005	0.78170	**0.00520**	0.70790	0.24580	0.75995	0.96405	0.25815	0.08190	0.70330	0.16685	0.17405	0.18010

AMOVA results, presented in Table 3, reveal that irrespective of any grouping, 2.38% of variation is attributable to differences among populations, while 11.6% of variation result from differences among the individuals within populations. The corresponding F_ST_ value of 0.02379 indicates a low degree of genetic differentiation, among the studied groups, which might probably be attributed to the recent formation of the different factions from a common ancestral group. Two important factors that might possibly have played a key role in the genetic differentiation of Adi are: fission due to inter-tribal conflicts and relative geographic isolation of the formed contemporary Adi sub-tribes [28]–[30]. So AMOVA analyses were performed to understand the relative influence of both these factors towards the genetic differentiation of Adi (Table 3). The grouping of populations based on their geophysical location (F_SC_: 0.02885) as well as their ethno-history (F_SC_: 0.02743) did not reveal any significant differences among the groups. In both cases, the variation among the populations and within the groups was around 2.8% and among the individual within the populations was around 11.6% as in case of the single group analysis. The variation within individuals was found to be around 86% at different levels of analyses.

**Table 3:** Genetic differentiation of Adi populations based on AMOVA

Grouping	Adi Populations in group	Source of Variation	Percentage of Variation	Fixation indices
Single group	Pasi-Upper, Pasi-Lower, Minyong, Panggi, Komkar, Padam	Among populations	2.38	F_ST_ : 0.02379
		Among Individuals within populations	11.57	
		Within Individuals	86.05	
Two groups based on geophysical location	(Pasi-Lower, Padam, Minyong) *vs* (Pasi-Upper, Komkar, Panggi)	Among groups	−0.81	F_SC_: 0.02885 F_CT_: −0.00806
		Among populations within groups	2.91	
		Among Individuals within populations	11.61	
		Within Individuals	86.29	
Three groups based on ethno-history	(Panggi-Komkar) *vs* (Padam-Minyong) *vs* (Lpasi-Upasi)	Among groups	−0.48	F_SC_ : 0.02743 F_CT_: −0.00481
		Among populations within groups	2.76	
		Among Individuals within populations	11.59	
		Withim individual	86.13	

**Figure 2:**
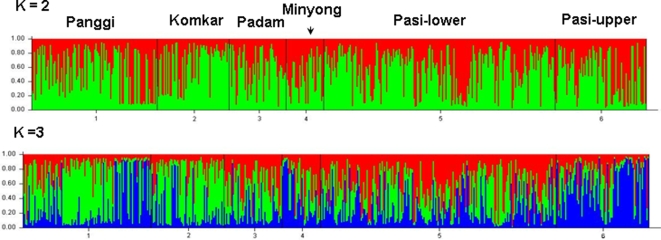
Bar plot estimation figures of six Adi sub-tribes, inferred from the STRUCTURE analysis.

To understand the extent of sub structuring among the Adi sub-tribes we have performed structure analysis with different values of *K.* Simulation summary for *K* = 2 and *K* = 3, including the logarithm of estimated probability of data (Ln Prob) values, values of proportion of membership of each pre-defined populations in each of the two or three clusters and the corresponding α values are given in Table 4. The pattern of sub structuring among the studied sub-populations is depicted in Figure 2. The log probability values and the membership proportions of each group, during different simulations, show no clear sub-structure among the Adi sub-tribes. In case of *K* = 2 and *K* = 3, Adi Komkar show higher proportion of membership (0.613 and 0.471) to cluster 1. Adi Pasi-Lower shows higher values of membership proportion (0.451) in case of clusters 2, when *K* = 3. The extent of genetic differentiation do not differ in case of K = 3 (mean value of α = 0.894) and *K* = 2 (mean value of α = 0.754) categories. The pattern depicted in Figure 2 (both for *K* = 2 and *K* = 3) shows no clear indication of sub-structuring among the six Adi sub-populations.

**Table 4:** Membership Proportions of each pre-defined six Adi populations in each of K clusters and logarithm of estimated probability of data.

Admixture Model with Correlated Allele Frequencies					
Simulations of data for the six Adi population					
	K = 2		K = 3		
Inferred Clusters	1	2	1	2	3
Panggi	0.567	0.463	0.389	0.368	0.243
Komkar	0.613	0.387	0.471	0.283	0.247
Padam	0.559	0.441	0.370	0.262	0.368
Minyong	0.451	0.549	0.288	0.344	0.367
Pasi- Upper	0.550	0.450	0.332	0.281	0.387
Pasi- Lower	0.469	0.531	0.267	0.451	0.282
Ln Prob	−25343.3		−2523.8		
α value	0.7539		0.8944		

(Ln Prob) for each assumed K

### Genetic affinity among the studied populations

**Figure 3:**
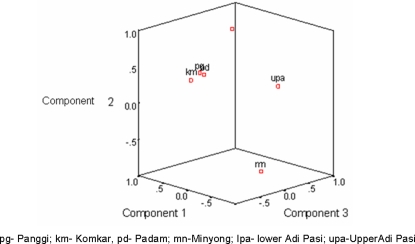
PCA plot, based on D_A_ distance, of the 6 studied Adi sub-groups.

The pattern of clustering and the genetic affinity between the six Adi sub-tribes are shown in the D_A_-NJ phylogenetic trees (supplementary Figure S1) and the PCA plot (Figure 3). The studied populations depict a single close cluster of four populations (Panggi, Komkar, Padam and Pasi-Lower), the remaining two populations, *viz.,* Adi Pasi-Upper and Adi Minyong separating away from the others. The PCA plot also show a similar pattern of clustering, substantiating the pattern obtained from the dendrogram, with the exception of Adi Pasi-Lower which was distantly located from the Panggi-Komkar-Padam cluster.

### Genetic relationship of Adi with other populations

#### a) Tibeto-Burman speaking populations of India

**Figure 4:**
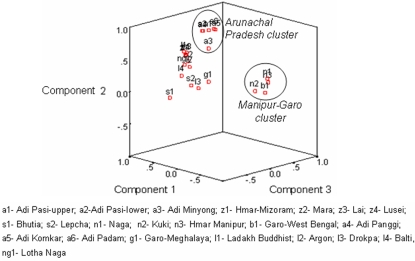
PCA plot, based on D_A_ distance, of the 22 Tibeto- Burman populations of India.

The D_A_-NJ phylogenetic tree, depicting the genetic relationship of Adi sub-populations with the sixteen neighboring Tibeto-Burman speaking populations of north and north-east India, is shown in supplementary Figure S2 and the corresponding PCA plot is depicted in Figure 4. Overall, the geographically proximate Tibeto-Burman populations tend to cluster together. The phylogeny exhibits 3 distinct major clusters. Cluster I consists of two sub-clusters, where the first sub-cluster, *Ladakh-Sikkim sub-cluster,* includes 4 populations from Ladakh [Ladakh Buddhist, Argon, Drokpa and Balti] and 2 populations from Sikkim [Bhutia and Lepcha]. Lotha Naga of Nagaland is found to be an outlier to the Ladakh populations. The second sub-cluster, *Mizoram sub-cluster*, comprise completely of the four tribal populations of Mizoram [Hmar, Mara, Lai and Lusei].

Cluster II (*Arunachal cluster*) consist exclusively of the six Adi sub-groups which retained their previous clustering pattern (Figures 2 and 3) and thereby revealing their distinct identity. The third major cluster (*Manipur-Garo cluster*) includes three populations from Manipur [Hmar, Naga and Kuki] along with the distantly located Garo of West Bengal. Garo of Meghalaya stands out as an outlier to this cluster. The corresponding PCA plot (Figure 4) reveal similar clustering pattern as observed in supplementary Figure S2, but the clusters 1 and 2 is not as clearly distinct as it was in the phylogeny. The third cluster, *Manipur-Garo cluster,* however retains their distinctness.

#### b) Populations of East and Southeast Asia

The D_A_-NJ phylogeny (supplementary Figure S3) comprising of the Tibeto-Burman speakers of India (including Adi) along with the linguistically diverse but morphologically similar populations of East and Southeast Asia, show geography based clustering of the analyzed populations, but the branching of the clusters are not distinct at the root. However, the sub-branching shows clear clustering of regional populations and at least 4 major clusters can be seen in the dendrogram. One cluster consist of populations from East Asia [4: Han Chinese- North-East, Chinese-East, Japanese and Korean] and Manipur of India [3: Hmar, Kuki and Naga] along with Garo of West Bengal. Another major cluster comprise of populations from Southeast Asia [7: Thai, Vietnamese, Filipinos, Malaysian Malays, Malaysian Singaporean, Malaysian Javanese and Indonesian] and Ladakh and Sikkim of India [3: Drokpa, Balti and Bhutia]. Yet another cluster includes chiefly the Tibetan populations [4: Naqu, Changdu, Lassa and Chinese Tibetan] along with 2 populations of Ladakh [Argon and Ladakh Buddhist], 3 populations of Nepal [Sherpa, Non-Sherpa and Nepalese], Lotha Naga of Nagaland, Lepcha of Sikkim and Bhutanese. One more cluster consist of populations from Arunachal Pradesh [six Adi sub-groups], Mizoram [4: Hmar, Mara, Lai and Lusei], Luoba of Tibet, Nepali of Sikkim and Garo of Meghalaya. The remaining populations [7: mainly from different geographical regions of China] are sparsely located in the phylogeny. The corresponding PCA plot (figure not shown) of this dataset did reveal similar clustering pattern.

## Discussion

Adi tribe comprises of several sub-tribes settled in relative geophysical isolation since several generations. They exhibit socio-cultural as well as linguistic diversity coupled with wide variation in subsistence pattern (ranging from hunting-gathering to settled agriculture). There were very few sporadic biological studies among some sub-tribes of Adi [18]–[19], [21]–[24], [32]–[36] and several of the isolated sub-tribes are yet to be investigated. This is perhaps the first ever comprehensive molecular genetic study attempted to investigate the genetic affinity and diversity among the sub-tribes of Adi and their relationship with other Tibeto-Burman tribes of India and the populations of East and Southeast Asia.

The results of the allele frequency variation at 15 STR loci reveal the underlying microsatellite diversity among the studied sub-tribes of Adi. The extent of deviation of the studied loci from HWE, among Adi, differs at the sub-structural level that is indicative of their unique population structure. For instance, Pasi-Upper and Panggi deviate from HWE at maximum number of loci (6 and 4 respectively) whereas Minyong show deviation at only one locus and Padam shows none. This scenario in case of Pasi-Upper and Panggi populations is probably due to their small size and relative isolation in remote Upper Siang hilly regions. In contrast, least deviation in Minyong (one locus) and absence of deviation in Padam might be the resultant of their comparative large population size distributed over plain East Siang regions, in proximity to the urban area.

The least average heterozygosity value among Panggi (∼74%) might be explained by their small size, strategic location and preferential marriage practices among clans prohibiting external gene flow [37]. Strikingly, the maximum average heterozygosity value (∼78%) was observed among Pasi-Upper in spite of their small population size and relative isolation. The results obtained from the exact test of population differentiation, for the 15 STR loci, also show wide diversity among the Adi sub-tribes. The least significant difference between Adi Padam and other sub-populations (at almost 7 loci) might be due to the fact that the other sub-tribes have formed from the larger Padam tribe, one of the earliest settlers of the region; however this requires further validation.

According to the folklore tradition of Adi, formation of their sub-groups was guided by fission-fusion process as a result of inter tribal war fares in the recent past. The above ethno-historical information is supported by the low average G_ST_ value (2.34%) among Adi which is an indication of the low degree of genetic differentiation among the sub-groups. This low degree of differentiation among the sub-groups is further substantiated by the clustering pattern obtained from the PCA plot and phylogeny, where all the sub-tribes form a single close cluster.

A close cluster of Komkar and Panggi sub-tribes, observed in the PCA plot and the phylogenetic tree, corroborates with their geographic proximity and also the clustering of Padam with this group yields further support to the ethno-historical account that Komkar and Panggi sub-groups were formed from the larger Padam group [26], [29]–[30]. The separation of Pasi-Upper and Pasi-Lower in the phylogeny, despite belonging to the same ancestral group, could be the consequence of the migration of a few close kin-groups from their ancestral population of Adi Pasi at the Upper Siang district to the plain areas at the East Siang district. As a result of isolation, there have been changes in the marriage patterns leading to higher endogamy among the Adi Pasi-Upper as against the inter-tribal marriages and low endogamy among the Adi Pasi-Lower.

AMOVA results also show least genetic differentiation among the sub-tribes of Adi. The low F_SC_ values (around 2.7%) irrespective of ethno-historical or geographical grouping of the populations suggest that the formation of the sub-populations was a recent phenomenon and that the ethno-history and geography had less influence on the overall genetic make up of the populations. So in spite of the socio-cultural, geographic and linguistic diversity, Adi sub-groups remain genetically less differentiated. However, this observation needs to be speculated as the increase in the number of samples and the microsatellite loci might contradict the above observation. The STRUCTURE analysis also support the findings of AMOVA, wherein no clear sub-structuring was observed among the Adi sub-populations (for both K = 2 and K = 3 runs). Overall, the low average G_ST_ values, close clustering in PCA plot and phylogeny, low F_ST_ and F_SC_ values of AMOVA and absence of discrete sub-structuring among Adi sub-tribes support their recent formation from a common ancestral group.

The phylogenetic analyses of Adi and other Tibeto-Burman groups show geography based clustering. Tibeto-Burman tribes of India are supposed to have arrived in several waves of migrations during different time periods from eastern, southeastern and central Asian regions and have localized in various geographical regions of the subcontinent [1], [5], [38]. The geophysical nature of the terrain especially of mountain valleys and rivers in different parts of sub-Himalayan mountain ranges might have acted as geographical barriers and posed hurdles for migration preventing gene flow from other regions and from other populations as well. This should have resulted in regional genetic differentiation of populations in their respective geographical regions. This expectation is validated by the results obtained from the phylogenetic tree of 22 Tibeto-Burman populations which shows the formation of clusters based on the geographic proximity of the populations. For example, the six subpopulations of Adi tribe inhabiting the Siang river valleys in central Arunachal Pradesh all cluster together. Similarly the tribes from Mizoram, Manipur, Sikkim and Ladakh from wide geographical regions form separate clusters. A deviation observed, from other studies as well, is the clustering of Garo of West Bengal and Garo of Meghalaya along with the Manipur tribes, though the Garo tribes are geographically separate from the tribes of Manipur. This probably suggests their common genetic affinity with the Manipur tribes and possible common migration history of these populations and the Manipur tribes. This further confirms the preliminary results of our earlier microsatellite study on Adi Pasi-Lower and other Tibeto-Burman populations of India [24].

The inclusion of populations from East and South-east Asia in the phylogenetic analyses reveals the clustering of the Luoba ethnic group of Tibet with the Adi groups of Arunachal Pradesh. According to the ethnologue information, Luoba Tibetan (Boga'er Luoba), categorized under the North-Assam branch of the Tibeto-Burman sub linguistic family, is also alternatively referred to as Adi/Abor and is supposed to have been derived from the ‘Tani’ group, the putative ancestral population of Adi. They are located in southern fringes of central Tibetan region, which is adjacent to the Upper Siang district of Arunachal Pradesh. The clustering of Luoba with Adi further supports the ethno-historical accounts of their putative common origin.

The fifty populations from East and Southeast Asian countries along with the Adi and other Indian Tibeto-Burman populations show an interesting pattern of clustering. Some Tibeto-Burman populations of India (e.g. Adi tribes of Arunachal Pradesh, populations from Ladakh, Mizoram, Sikkim, and Garo of Meghalaya) get clustered with the Tibetan populations from Tibet and China whereas some others (e.g. Drokpa, Balti of Ladakh and Bhutia of Sikkim) cluster along with Southeast Asian populations. All the morphologically similar populations, irrespective of their linguistic affiliation, cluster together possibly with respect to their geography and ethno-historical account of migration.

Overall, Adi and other Tibeto-Burman speaking populations of India are regionally well differentiated and exhibit genetic affinity with the neighboring populations of East/Southeast Asia, based on their shared ethno-history. However, a clearer picture will possibly emerge from the analysis of increased number of informative genetic markers and from the uniparental markers like mitochondrial DNA and Y chromosome.

## Materials and Methods

### Populations

Blood samples were collected with the permission of the District Circle Officer, (DCO), East and Upper Siang districts, and approval of the ‘*Gaon Burah’* (village head) of each village of the studied populations. Prior informed consent were obtained verbally from 836 healthy voluntary participants belonging to five sub-tribes [Pasi, Minyong, Padam, Panggi and Komkar] of the Adi tribe, distributed at different villages in East Siang (low altitude) and the Upper Siang (high altitude) districts of Arunachal Pradesh, north east India (see Introduction for further details on Adi tribe). Among the five populations of Adi, Pasi was sampled from both East and Upper Siang districts and the two sampled groups were treated separately as Adi Pasi-Lower and Adi Pasi-Upper. Prior approval for the study was obtained from the ‘Indian Statistical Institute Review Committee for Protection of Research Risk to Humans’. The five sub-tribes of Adi selected for this study, were analyzed for a set of 15 autosomal STR (microsatellites) markers to unravel the genetic structure and affinity.

Further, to understand the genetic relationships between Adi sub-tribes and other neighboring Tibeto-Burman speaking populations, the generated autosomal STR data of Adi was compared with the published allele frequency data (for the nine common loci) of other sixteen Tibeto-Burman speaking populations from north (Ladakh) and northeast (Mizoram, Manipur, Sikkim, Nagaland and Meghalaya) India [39]–[44]. Also the observation that Tibeto-Burman speakers of the Indian subcontinent share similar physical features with that of the East and Southeast Asian populations instigated us to comprehend the genetic status of Tibeto-Burman speakers of India (including Adi) amidst the linguistically diverse but physically akin populations of East/Southeast Asia. So we compared the populations of north and northeast India, based on the available allele frequency data of nine common STR loci, along with that of East/Southeast Asia [45]–[68]. Due to the unavailability of the genotype data for the studied reference populations, we had no other option but to restrict our analyses based on the available allele frequency data. Details of all the studied populations, their sample size, ethnic and linguistic affiliations, geographical locations, subsistence patterns and their literature sources are given in the supplementary Table S1.

### DNA isolation and microsatellite typing

High molecular weight DNA was isolated, from the collected blood samples of Adi sub-tribes, using the standard phenol/chloroform method [69]. One to 10 ng of individual DNA template were amplified for fifteen tetranucleotide repeat loci (D5S818, FGA, D8S1179, D21S11, D7S820, CSF1PO, D3S1358, THO1, D13S317, D16S539, D2S1338, D19S433, vWa, TPOX, and D18S51) on Gene-Amp PCR 9700 thermal cycler (Applied Biosystems, Foster City) by using the AmpF*I* STR® Identifiler kit (Applied Biosystems, Foster City) according to manufacturer's instructions. While the amplified products of Pasi and Minyong sub-tribes were separated on a 4% polyacrylamide gel using the ABI Prism 377 automated DNA sequencer (Applied Biosystems), the amplified fragments of Panggi, Komkar and Padam sub-tribes were separated and detected using the ABI Prism® 3100-Avant Genetic Analyzer (Applied Biosystems, Foster City). The resultant data was then analyzed using GeneScan™ Analysis Software (Version 3.7) and the allele designations were done with Genotyper™ DNA Fragment Analysis Software (Version 3.7) (Applied Biosystems, Foster City). The laboratory experiments were carried out following all the quality control measures.

### Statistical Analyses

The allele frequencies of the 15 STR loci were calculated, from the obtained genotype data of the Adi sub-tribes, using the DNATYPE software [70]. The observed heterozygosity (*h*) at each locus and the probability of homozygosity (*P*) were estimated to evaluate the extent and magnitude of genetic diversity among the sub-groups of Adi. Also, likelihood ratio test (LR) and the exact test (ET) were performed to test the possible divergence of each locus from the Hardy-Weinberg Expectations (HWE) [71]–[72]. Based on the allele frequencies of the 15 autosomal STR loci of six Adi populations, the locus-wise genetic diversity (G_ST_) [73]-[74] and the population-wise average heterozygosity were estimated to understand the degree of genetic differentiation and the within-population heterogeneity respectively. Locus-wise exact test of population differentiation, using Arlequin 3.01, was performed to analyze the extent of genetic diversity at the studied loci among the six Adi sub-tribes [75].

To understand the genetic relatedness between the six studied Adi populations; pair-wise genetic distances using the modified Cavalli-Sfroza distance (D_A_) and the standard genetic distance (D_ST_) measures of Nei et al., [76] were computed using the software DISPAN [77]. Subsequently, the conventional rectangular form of two phylogenetic trees: the unweighted pair group method with arithmetic mean (UPGMA) tree and neighbor-joining (NJ) tree were constructed based on the D_A_ and D_ST_ distance measures by employing the software DISPAN. To check for the reliability and consistency of the clustering pattern of the obtained dendrograms, a total of 1000 and also 10,000 bootstrap replications were separately performed. In order to further explore the topology of the obtained phylogenetic trees including the positions and lengths of the branches, branching patterns as well as the cluster formation, the radiation form of the trees were also constructed using the phylogenetic software Mega v3.1 [78]. Since D_A_ distance measure is the most efficient for obtaining correct phylogenetic trees under various evolutionary conditions and also is least affected by small size [79], and because UPGMA and the NJ phylogenies depict a similar pattern of relationship between the populations, our discussions are based only on the D_A_–NJ trees.

To characterize the clustering trends exhibited by these studied populations, the data dimensionality was reduced by performing a covariance analysis between factors [Principle Component Analysis (PCA)]. This analysis was performed based on the D_A_ distance matrix, of the six Adi sub-groups, using SPSS software (Version 11.0), Chicago, IL, USA. The PCA plot further substantiates the dendrogram clustering method, and especially when bootstrap values of the dendrogram are considerably low, the similar clustering in both the PCA plot and the dendrogram indicates the consistency of the results obtained.

In order to investigate the genetic variation within and between the sub-populations of Adi, Analysis of Molecular Variance (AMOVA) was performed using Arlequin 3.01 [75]. Also, the significance of the AMOVA values was estimated by use of 10,000 permutations. Three levels of analyses were performed, wherein at the first level the six Adi sub-groups [Pasi-Upper, Pasi-Lower, Minyong, Panggi, Komkar and Padam] were considered as a ‘single group’. At the second level, the six Adi populations were categorized into ‘two groups’ based on their geophysical locations [Group 1: Padam, Minyong, Pasi-Lower; Group 2: Panggi, Komkar, Pasi-Upper]. The populations of group 1 (Padam, Minyong and Pasi-Lower) are located at the lower plains of the Siang river valley and geographically separate from the populations of group 2 (Panggi, Komkar, Pasi-Upper) which, on the contrary, are isolated and settled at the higher mountain ranges. At the third level of analyses, three groups were constructed based on the ethno-historical information of the sub-groups [Group 1: Panggi and Komkar; Group 2: Padam and Minyong; Group 3: Pasi-Upper and Pasi-Lower].

To obtain a vivid insight into the sub structuring among Adi sub-tribes, a model-based clustering method was employed, using genotype data consisting of unlinked markers, as implemented in *Structure* 2.1 program [80]. The program was performed by using 100,000 MCMC replications after a 20,000 burn-in length. Simulations were done with different values of *K* (from 1 to 5) under the assumption of admixture model and correlated allele frequencies among populations. Each run was carried out several times to ensure consistency of the results.

In addition to the above analyses performed on Adi populations, we also conducted the comparative analyses of Adi sub-groups with sixteen Tibeto-Burman speaking populations of north and northeast India and also with other neighboring East and Southeast Asian populations that share similar physical features with that of Adi. Phylogenetic analysis (as described above) as well as Principle Component Analysis, were performed on these populations, based on the available allele frequency data, to understand their underlying genetic affinity and also to obtain a better clarity of the genetic status of Adi populations with the Tibeto-Burman speaking regional populations and linguistically diverse other global populations of East/Southeast Asia.

## Supporting Information

Table S1 Sample size, geographical distribution, linguistic affiliation and the subsistence pattern of the studied populations.
(0.10 MB DOC)

Figure S1 DA-NJ phylogenetic trees, based on 15 loci, depicting the genetic relationship between the Adi sub-groups.
(0.05 MB TIF)

Figure S2 DA- NJ phylogenies, based on 9 loci, depicting the genetic relationship between 22 Tibeto-Burman populations of India.
(0.09 MB TIF)

Figure S3 DA- NJ phylogenies, based on 9 loci, depicting the genetic relationship between 50 populations of India and East/Southeast Asia.
(0.18 MB TIF)
