# Supplementary material for: A Microsatellite Guided Insight into the Genetic Status of Adi, an Isolated Hunting-Gathering Tribe of Northeast India
Source: PLoS One. 2008 Jul 2;3(7):e2549. doi: 10.1371/journal.pone.0002549 (PMC2435608; doi:10.1371/journal.pone.0002549)
Supplement: Table S1 — Sample size, geographical distribution, linguistic affiliation and the subsistence pattern of the studied populations. (0.10 MB DOC) [file pone.0002549.s001.doc]

| **Name of the population**  **Table S1. Sample size, geographical distribution, ethnic and linguistic affiliations and the subsistence patterns of the studied populations** | **Sample Size** | **Geographic Distribution** | **Linguistic Affiliation*** | **Traditional Occupation** | | **Microsatellite Data Source** | |  | |
| --- | --- | --- | --- | --- | --- | --- | --- | --- | --- |
|  |  |  | **North and North-East India** |  | |  | |  | |
| AdiPasi (upper) | 121 | Arunachal Pradesh | ST, TB, North-Assam, Tani | | Hunting-Gathering, Shifting Cultivation | | Present Study | | |
| AdiPasi (lower) | 203 | Arunachal Pradesh | ST, TB, North-Assam, Tani | | Hunting-Gathering, Shifting Cultivation | | Present Study | | |
| Adi Minyong | 33 | Arunachal Pradesh | ST, TB, North-Assam, Tani | | Hunting-Gathering, Shifting Cultivation | | Present Study | | |
| Hmar-Mizoram | 80 | Mizoram | ST, TB, Kuki-Chin-Naga, Kuki-Chin, Central | | Shifting Cultivation | | Maity et al. 2003 | | |
| Mara | 90 | Mizoram | ST, TB, Kuki-Chin-Naga, Kuki-Chin, Southern | | Shifting Cultivation | | Maity et al. 2003 | | |
| Lai | 92 | Mizoram | ST, TB, Kuki-Chin-Naga, Kuki-Chin, Central | | Shifting Cultivation | | Maity et al. 2003 | | |
| Lusei | 92 | Mizoram | ST, TB, Kuki-Chin-Naga, Kuki-Chin, Central | | Shifting Cultivation | | Maity et al. 2003 | | |
| Bhutia | 75 | Sikkim | ST, TB, Himalayish, Tibeto-Kanauri, Tibetic, Tibetan, Southern | | Shifting Cultivation | | Kashyap et al. 2002 | | |
| Lepcha | 48 | Sikkim | ST, TB, Himalayish, Tibeto-Kanauri, Lepcha | | Agriculture | | Kashyap et al. 2002 | | |
| Naga | 106 | Manipur | ST, TB, Kuki-Chin-Naga, Naga, Tangkhul | | Shifting Cultivation | | Chattopadhyay et al. 2001 | | |
| Kuki | 105 | Manipur | ST, TB, Kuki-Chin-Naga, Kuki-Chin, Northern | | Shifting Cultivation | | Chattopadhyay et al. 2001 | | |
| Hmar-Manipur | 101 | Manipur | ST, TB, Kuki-Chin-Naga, Kuki-Chin, Central | | Shifting Cultivation | | Chattopadhyay et al. 2001 | | |
| Garo-West Bengal | 110 | West Bengal | ST, TB, Jingpho-Konyak-Bodo, Konyak-Bodo-Garo, Bodo-Garo, Garo | | Shifting Cultivation | | Chattopadhyay et al. 2001 | | |
| Adi Panggi | 110 | Arunachal Pradesh | ST, TB, North-Assam, Tani | | Hunting-Gathering, Shifting Cultivation | | Present Study | | |
| Adi Komkar | 63 | Arunachal Pradesh | ST, TB, North-Assam, Tani | | Hunting-Gathering, Shifting Cultivation | | Present Study | | |
| Adi Padam | 50 | Arunachal Pradesh | ST, TB, North-Assam, Tani | | Hunting-Gathering, Agriculture | | Present Study | | |
| Garo-Meghalaya | 128 | Meghalaya | ST, TB, Jingpho-Konyak-Bodo, Konyak-Bodo-Garo, Bodo-Garo, Garo | | Shifting Cultivation, Agriculture | | Langstieh et al. 2004 | | |
| Ladakh Buddhist | 156 | Ladakh | ST, TB, Himalayish, Tibeto-Kanauri, Tibetic, Tibetan, Western, Ladakhi | | Priesthood | | Trivedi et al. 2002 | | |
| Argon | 51 | Ladakh | ST, TB | | Trade and Commerce | | Trivedi et al. 2002 | | |
| Drokpa | 33 | Ladakh | ST, TB | | Trade and Agriculture | | Trivedi et al. 2002 | | |
| Balti | 67 | Ladakh | ST, TB, Himalayish, Tibeto-Kanauri, Tibetic, Tibetan, Western | | Trade and Agriculture | | Trivedi et al. 2002 | | |
| Lotha Naga | ~ 200 | Nagaland | ST, TB,Kuki-Chin-Naga, Naga | | Agriculture | | Mastana et al. 2007 | | |
| Nepali | 220 | Sikkim | Indo-European, Indo-Iranian, Indo-Aryan, Northern zone, Eastern Pahari | | Agriculture | | Kashyap et al. 2002 | |  |
| **Name of the population** | **Sample Size** | **Geographic Distribution** | **Linguistic Affiliation*** | | **Traditional Occupation** | | **Microsatellite Data Source** | |  |

**East and South-east Asia**

| Chinese Tibetan | 850 | Qinghai Province, China | ST,TB Himalayish, Tibeto-Kanauri,  Tibetic, Tibetan, Northern | Agriculture, Animal husbandry and Industry | | | Yan et al. 2007 | |  |
| --- | --- | --- | --- | --- | --- | --- | --- | --- | --- |
| Lassa Tibetan | 196 | Lassa of Tibet, SW China | ST,TB Himalayish, Tibeto-Kanauri,  Tibetic, Tibetan, Central | | Agriculture, Animal husbandry and Industry | | Li et al. 2006 | | |
| Chinese | 200 | Sichuan area, W China | ST, Chinese | Agriculture, Forestry, Mining and Industry | | | Zhang et al. 2006 | |  |
| Han Chinese | ~122 | Min Nan area, SE China | ST, Chinese | -- | | | Hu et al. 2005 | |  |
| Han Chinese | 203 | Shaanxi Province, NW China | ST, Chinese | Agriculture and Animal husbandry | | | Wang et al. 2005 | |  |
| Han Chinese | 200 | Jilin Province, NE China | ST, Chinese | Agriculture and Animal husbandry | | | Yang et al. 2005 | |  |
| Chinese | 100 | China, East | ST, Chinese | Agriculture and Animal husbandry | | | Gao et al. 2005 | |  |
| Thai | 210 | Thailand | Tai-Kadai, Kam-Tai, Be-Tai, Tai-Sek, Tai, Southwestern, East Central, Chiang Saeng | Agriculture, Forestry and Fishing | | | Rerkamnuaychoke et al. 2006 | |  |
| Japanese | 650 | Japan | Japanese, Japanese | Agriculture and fishing | | | Tie et al. 2006 | |  |
| Korean | 231 | Korea | Language isolate | Agriculture and Forestry | | | Kim et al. 2003 | |  |
| Malays | 212 | Malaysia | Austronesian, Malayo-Polynesian,  Malayic, Malayan, Local Malay | Agriculture and fishing | | | Lzuan et al. 2005 | |  |
| Chinese | 234 | Malaysia | ST, Chinese | -- | | | Lzuan et al. 2005 | |  |
| Han Chinese | 208 | China, Central | ST, Chinese | Agriculture and Animal husbandry | | | Zeng et al. 2007 | |  |
| Filipinos | 106 | Philippines | Austronesian, Malayo-Polynesian, Meso Philippine, Central Philippine, Tagalog | Agriculture and Industry | | | De Ungria et al. 2005 | |  |
| Taiwanese | 597 | Taiwan | ST, Chinese | Agriculture and Industry | | | Wang et al. 2003 | |  |
| Vietnamese | 178 | Hanoi area, Vietnam | Austro-Asiatic, Mon-Khmer,  Viet-Muong, Vietnamese | Agriculture, Fishing and Forestry | | | Shimada et al. 2002 | |  |
| Bhutanese | 936 | Bhutan | ST, TB, Himalayish, Tibeto-Kanauri,  Tibetic, Tibetan, Southern | Agriculture, Fishing and Forestry | | | Kraaijenbrink et al. 2006 | |  |
| Nepalese | 953 | Nepal | Indo-European, Indo-Iranian, Indo-Aryan, Northern zone, Eastern Pahari | Agriculture | | | Kraaijenbrink et al. 2006 | |  |
| Sherpa | 105 | Nepal | ST, TB, Himalayish, Tibeto-Kanauri,  Tibetic, Tibetan, Southern | Agriculture, Animal husbandry and trade | | | Ota et al. 2007 | |  |
| Non-Sherpa | 111 | Kathmandu valley, Nepal | ST, TB, Himalayish, Tibeto-Kanauri,  Tibetic, Tamangic | Agriculture | | | Ota et al. 2007 | |  |
| Changdu Tibetan | 100 | Tibet Province, China | ST, TB, Himalayish, Tibeto-Kanauri,  Tibetic, Tibetan, Northern | Agriculture, Animal husbandry and Industry | | Longli et al. 2007 | |  | |
| Naqu Tibetan | 118 | Tibet Province, China | ST, TB, Himalayish, Tibeto-Kanauri,  Tibetic, Tibetan, Northern | Agriculture, Animal husbandry and Industry | | Longli et al. 2007 | |  | |
| Indonesian | 105 | East of Java Islands | Austronesian, Malayo-Polynesian,  Javanese | Agriculture | | Dovashi et al. 2005 | |  | |
| Malaysian Javanese | 218 | Malaysia | Austronesian, Malayo-Polynesian,  Javanese | Trade | | Othman et al. 2004 | |  | |
| Malay Singaporean | 161 | Singapore | Austronesian, Malayo-Polynesian,  Malayic, Malayan, Local Malay | -- | |  | |  | |
| Chinese Singaporean | 184 | Singapore | ST, Chinese | -- | |  | |  | |
| Luoba | 93 | Tibet, SW China | ST, TB, North-Assam, Tani | Agriculture and Hunting | | Long and Li, 2005 | |  | |
|  |  |  |  |  | |  | |  | |

* ST- Sino-Tibetan, TB – Tibeto-Burman
