# Supplementary figures and images for: A Microsatellite Guided Insight into the Genetic Status of Adi, an Isolated Hunting-Gathering Tribe of Northeast India
Source: PLoS One. 2008 Jul 2;3(7):e2549. doi: 10.1371/journal.pone.0002549 (PMC2435608; doi:10.1371/journal.pone.0002549)

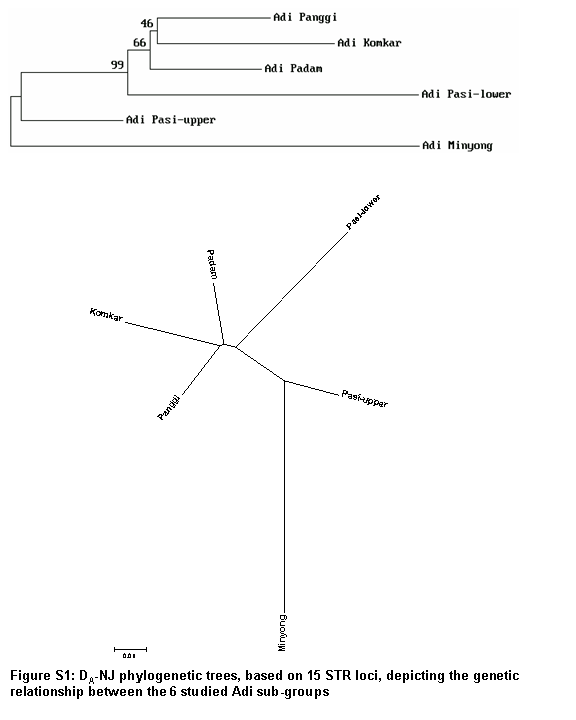

Supplement: Figure S1 — DA-NJ phylogenetic trees, based on 15 loci, depicting the genetic relationship between the Adi sub-groups. (0.05 MB TIF) [file pone.0002549.s002.tif]

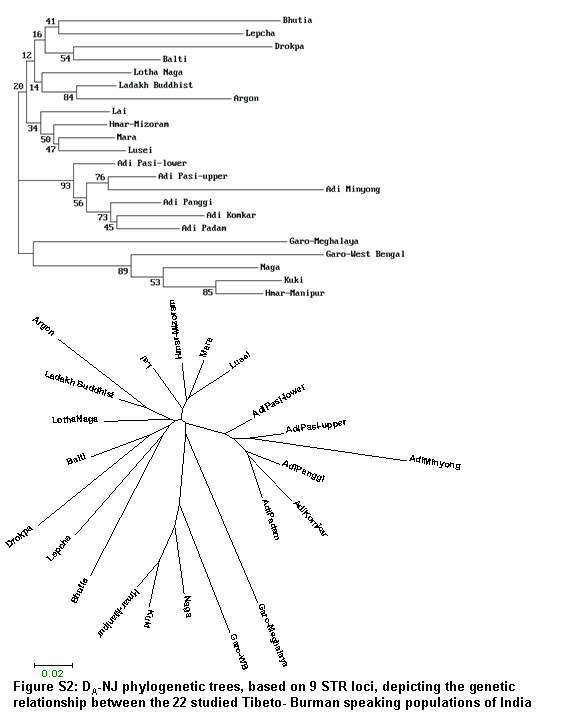

Supplement: Figure S2 — DA- NJ phylogenies, based on 9 loci, depicting the genetic relationship between 22 Tibeto-Burman populations of India. (0.09 MB TIF) [file pone.0002549.s003.tif]

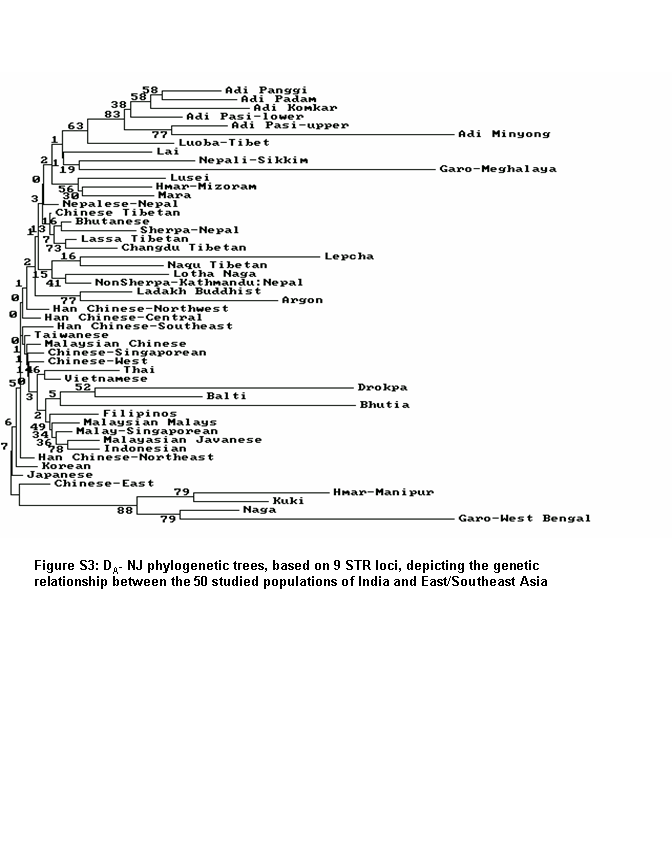

Supplement: Figure S3 — DA- NJ phylogenies, based on 9 loci, depicting the genetic relationship between 50 populations of India and East/Southeast Asia. (0.18 MB TIF) [file pone.0002549.s004.tif]
